# Comparing Population Patterns to Processes: Abundance and Survival of a Forest Salamander following Habitat Degradation

**DOI:** 10.1371/journal.pone.0093859

**Published:** 2014-04-09

**Authors:** Clint R. V. Otto, Gary J. Roloff, Rachael E. Thames

**Affiliations:** Department of Fisheries and Wildlife, Michigan State University, East Lansing, Michigan, United States of America; Universität Zurich, Switzerland

## Abstract

Habitat degradation resulting from anthropogenic activities poses immediate and prolonged threats to biodiversity, particularly among declining amphibians. Many studies infer amphibian response to habitat degradation by correlating patterns in species occupancy or abundance with environmental effects, often without regard to the demographic processes underlying these patterns. We evaluated how retention of vertical green trees (CANOPY) and coarse woody debris (CWD) influenced terrestrial salamander abundance and apparent survival in recently clearcut forests. Estimated abundance of unmarked salamanders was positively related to CANOPY (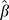 *_Canopy_*  = 0.21 (0.02–1.19; 95% CI), but not CWD (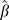 *_CWD_*  = 0.11 (−0.13–0.35) within 3,600 m^2^ sites, whereas estimated abundance of unmarked salamanders was not related to CANOPY (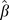 *_Canopy_*  = −0.01 (−0.21–0.18) or CWD (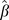 *_CWD_*  = −0.02 (−0.23–0.19) for 9 m^2^ enclosures. In contrast, apparent survival of marked salamanders within our enclosures over 1 month was positively influenced by both CANOPY and CWD retention (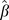 *_Canopy_*  = 0.73 (0.27–1.19; 95% CI) and 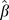 *_CWD_*  = 1.01 (0.53–1.50). Our results indicate that environmental correlates to abundance are scale dependent reflecting habitat selection processes and organism movements after a habitat disturbance event. Our study also provides a cautionary example of how scientific inference is conditional on the response variable(s), and scale(s) of measure chosen by the investigator, which can have important implications for species conservation and management. Our research highlights the need for joint evaluation of population state variables, such as abundance, and population-level process, such as survival, when assessing anthropogenic impacts on forest biodiversity.

## Introduction

Anthropogenic habitat degradation is a primary threat to global biodiversity [1], [2]. For example, greater than 30% of amphibian species worldwide are at risk of extinction from different forms of environmental degradation, with anthropogenic habitat degradation often cited as a leading cause of population declines [3]–[5]. One form of habitat degradation that negatively impacts forest dependent wildlife like some amphibians is timber harvesting [6]–[8]. Research shows that timber harvesting negatively affects forest amphibian abundance [9]–[11], but the population mechanism(s) that lead to these observed patterns in abundance are poorly understood [12], [13].

Most observational and experimental studies on amphibians and forestry use indices like species richness (counts of the number of species), relative abundance (counts of individuals within a species), or occurrence (counts of occupied sites) as response variables [14], [15]. Although these state variables are useful for inferring broad-scale impacts of environmental perturbations [16], [17], they have been criticized for failing to elucidate mechanisms of demographic change[13], [14], [18]. Indeed, research shows that patterns in amphibian counts may not reflect amphibian survival estimates [19]. Studies that directly assess the influence of habitat degradation on population vital rates such as survival, reproduction, and movement of organisms should yield greater inferential power than those that solely assess population indices [20]–[23]. However, demographic studies are often conducted at small spatial scales with limited replication, which may reduce the breadth of inference and applicability to broad-scale management [24]. Ideally, population patterns (counts) and processes (demography) should be jointly evaluated to better understand wildlife response to habitat degradation.

Few studies that assessed the impact of habitat degradation on terrestrial wildlife have combined broad-scale surveys with demographic research. We combined correlative and experimental approaches to investigate red-backed salamander (*Plethodon cinereus*) response to residual forest structure, such as coarse woody debris and vertical green trees, within recently harvested forests. These structures were purposefully retained to potentially ameliorate the negative effects of clearcutting on forest wildlife [25]. First, we studied how patterns in salamander abundance at two spatial scales (3,600 m^2^ and 9 m^2^) were related to retention of green trees (CANOPY) and coarse woody debris (CWD) within recent clearcuts in a managed, forested landscape. Second, we quantified how salamander apparent survival over 1 month in the summer was influenced by CANOPY and CWD. By focusing our study on the same species, forested areas, and disturbance type we were able to evaluate if abundance measurements collected at two spatial scales, and survival measurements, yielded similar inferences regarding amphibian response to forest management.

## Methods

### Study Species

Red-backed salamanders are a terrestrial, lung-less amphibian distributed in woodlands throughout eastern North America [26]. Terrestrial salamanders are recognized as critical components of forested ecosystems through their contribution to the detrital food web, forest biomass and may potentiality serve as indicators of forest health [27], [28]. Like all plethodontids, respiration in red-backed salamanders occurs cutaneously, which requires moist skin, making them susceptible to desiccation. As a result, the red-backed salamander has been utilized in many forest management studies and its negative response to clearcutting has been well documented [10], [11].

### Study Area

We conducted our study across a 560,000 ha area in the northwestern Lower Peninsula of Michigan, USA, in 2010-11. Our study occurred on state-owned forest lands that were managed for aspen (*Populus* spp.) production by the Michigan Department of Natural Resources (MDNR). The MDNR issues “Use Permits” for research conducted on state-owned lands. Use permits for our project were filed and approved consistent with MDNR expectations and are currently stored at the Cadillac and Traverse City, MI, field offices of the MDNR. In Michigan, aspen is typically harvested via clearcutting on a 40- to 60-year rotation. Within harvested stands (where a stand is defined as an area with homogenous vegetation and management focus) the MDNR implemented green-tree retention prescriptions to mitigate the negative effects of timber harvesting on wildlife [25]. These prescriptions called for retention of 3–10% of the pre-harvest green-tree basal area (*i.e.,* the cumulative surface area covered by a cross-section of tree stems at ground level), arranged throughout the stand as single leave-trees or aggregated into retention patches [29]. Harvested areas also contained varied amounts of CWD that was unequally distributed. Additional study area details can be found in [30]. For this study we focused on quantity of green-trees and CWD (i.e., how much), as opposed to characteristics of individual pieces (i.e., size class, decay state, species) because quantity is directly linked to the MDNR structural retention guidelines [29].

### Large-scale Abundance Data

All state-owned aspen stands within a four county area that were >8 ha in size and between 1 to 5 years post-harvest were potential candidates for sampling. We used a Geographic Information System (GIS; ArcGIS 9.1; Environmental Systems Research Institute, Redlands, CA) to overlay each aspen stand with a 60×60 m (0.36 ha) lattice and orthophotos from the 2010 National Agricultural Imagery Program (NAIP; http://www.mcgi.state.mi.us/mgdl) to digitize canopy cover of all retained green-trees within the sampling lattice for each forest stand. We assigned each cell of the lattice to a canopy cover group (>25%, 10–25%, and <10%) and randomly selected 40 cells from each group. We ensured that all selected cells were >200 m apart. We also selected 30 cells within 40- to 60-year-old aspen stands that were adjacent to our harvested stands. We eliminated 16 cells (13 harvested, 3 older) after initial field visits because the dominant cover-type was not aspen. Our final sample size for harvested cells was 107, with varying levels of green-tree canopy cover, and 27 for the 40–60 year-old cells. Hereafter, we refer to the subset of 60×60 m cells used for our study as sites.

Within each selected site we identified 33, 20×2 m transects that were oriented north to south and spaced ≥5 m apart. From the 33 transects we randomly selected 3 transects, with replacement, for salamander sampling. Selected transects were treated as spatial replicates for estimating salamander capture probability [31]. We sampled subunits (i.e., transects) with replacement to minimize estimation bias of the state-space models used for analysis [32]. We used spatial, as opposed to temporal, replication for sampling salamanders because it minimized the number of repeated visits to each site and reduced travel between sites. Furthermore, previous work shows that temporally replicated cover object searches often violate the “closure” assumption of the state-space models we used for analysis [30], [33]. Each transect was surveyed once unless it was selected with replacement, in which case it was surveyed again 12–16 days later. For each transect survey, one observer searched for salamanders under woody cover objects >4 cm diameter and >15 cm long. All woody cover objects consisted of downed logs from previous timber harvest or blow-down events. Observers tallied the number of woody cover objects they searched along each transect. We only included transects with >4 CWD objects of sufficient size in the analysis to ensure all transects had a minimum level of sampling effort. Site-level surveys were completed on the same day generally within 30 min. To assess variation in counts among transects within a site, we calculated a standard deviation for salamander counts at each site and then averaged the standard deviation across all sites.

We used counts of salamanders collected at each site and binomial mixture models [31] to estimate salamander abundance (N*_I_*) and detection probability (p). We hypothesized that salamander abundance would be lower in 1–5 year-old sites compared to 40–60 year-old sites (i.e., CONTROL covariate; Table 1). This hypothesis has been tested previously, and thus not a primary focus of our study [9], [10], [34]. We also hypothesized that salamander abundance at harvested sites would positively relate to structural retention, such as CANOPY and CWD. Although we stratified canopy cover into different categories during site selection, we treated CANOPY as a continuous variable (i.e., percent canopy cover) for all analyses. We considered models where salamander detection probability was held constant (p(.)) or varied as a function of CWD count (p(CWD*_j_*) along transect *j* at a site. As an exploratory analysis we included average daily temperature and daily precipitation as covariates on salamander detection probability for our 2 highest-ranking models. Although we standardized our salamander surveys to the spring and early summer when temperature and precipitation were conducive to salamander surface detection, we included these weather covariates to account for their potential effects on detection variation. Additional details regarding hypothesis and model development can be found in Text S1. We note that inferences for our large-scale study are limited to the proportion of salamander populations underneath or inside CWD objects, not the entire population of salamanders in the leaf litter or soil profile [35]. We assume that salamanders distributed underneath CWD on a given day are representative of the total salamander population. Our previous work suggests that this assumption is supported in recently harvested aspen stands [36].

**Table 1:** Ranking of candidate N-mixture (abundance  =  N) and Robust Design (survival  =  S) models for red-backed salamanders in harvested aspen stands in the northern Lower Peninsula of Michigan, USA, 2010–2011.

Model	Δ AIC*_c_* a	*w* a	*K* a	−2*l* a	CANOPY b	CWD b
Large-Scale Abundance (3,600 m^2^ sites)						
N(CANOPY +CONTROL), p(CWD)	0.00	0.36	5	558.8	0.21 (0.03–0.40)	
N(CWD + CANOPY + CONTROL), p(.)	1.18	0.20	5	559.9	0.21 (0.02–0.40)	0.23 (0.06–0.40)
N(CWD + CANOPY + CONTROL), p(CWD)	1.40	0.18	6	558.0	0.21 (0.03–0.40)	0.11 (−0.13–0.35)
N(CONTROL), p(CWD)	2.54	0.10	4	563.5		
Small-Scale Abundance (9 m^2^ enclosures)						
N(CONTROL), p(*t*)	0.00	0.36	5	440.8		
N(CONTROL), p(*t* + CWD)	0.94	0.23	6	439.0		
N(CWD + CONTROL), p(*t* + CWD)	2.50	0.10	7	437.8		0.20 (−0.19–0.58)
N(CWD + CONTROL), p(*t*)	2.65	0.10	6	440.7		−0.01 (−0.21–0.18)
Survival (9 m^2^ enclosures)						
S(CWD + CANOPY + CONTROL), p(*t*) = c(*t*)	0.00	0.56	7	1220.4	0.71 (0.26–1.17)	0.96 (0.50–1.42)
S(CWD + CANOPY + CONTROL), p(*t*) = c(*t*) + *b*	1.65	0.24	8	1219.1	0.67 (0.27–1.07)	0.85 (0.44–1.27)
S(CWD + CANOPY + CONTROL), p(*t* + CWD) = c(*t* + CWD)	2.90	0.13	8	1220.3	0.72 (0.26–1.18)	0.94 (0.45–1.43)
S(CWD + CANOPY + CONTROL), p(*t* + CWD) = c(*t* + CWD) + *b*	4.80	0.05	9	1219.1	0.67 (0.27–1.07)	0.86 (0.42–1.29)
S(CONTROL), p(*t* + CWD) = c(*t* + CWD) + *b*	15.48	0.00	5	1241.4		

a ΔAIC*_c_*  =  difference from the Akaike's Information Criterion (AIC) best model, adjusted for small sample size, *w*  =  AIC*_c_* model weight, *K*  =  no. of parameters, −2*l*  =  twice the negative log-likelihood.

b Beta estimates for abundance covariates CANOPY and CWD with 95% CI in parentheses.

### Small-scale Abundance and Survival Data

To quantify small-scale abundance (N*_i_*) and survival (S*_i_*) we selected 6 harvested stands that were sampled as part of our large-scale abundance study. These stands were selected to represent non-uniform variation in green tree retention levels. We overlaid a 30×30 m lattice and used a stratified (*i.e.*, >25%, 10–25%, and <10% canopy cover) random selection to identify 36 cells for study. Although our primary objective was to relate salamander survival to the structural characteristics of 1–5 year-old stands, we also selected nine cells within two, 40–60 year-old aspen stands that were adjacent to our 1–5 year-old stands as a basis for comparison. All cells were >50 m apart. Our selection of 40–60 year-old stands represents the near-maximum age class of aspen in our study area. We recognize that 40–60 year-old aspen stands do not represent high-quality habitat for terrestrial salamanders or provide ideal reference conditions for studying salamander survival [37]. We selected this age class of aspen because it represents a substantial portion of the deciduous cover type in this landscape and hence, in some areas, may be the only older deciduous cover type available for terrestrial salamanders.

**Figure 1:**
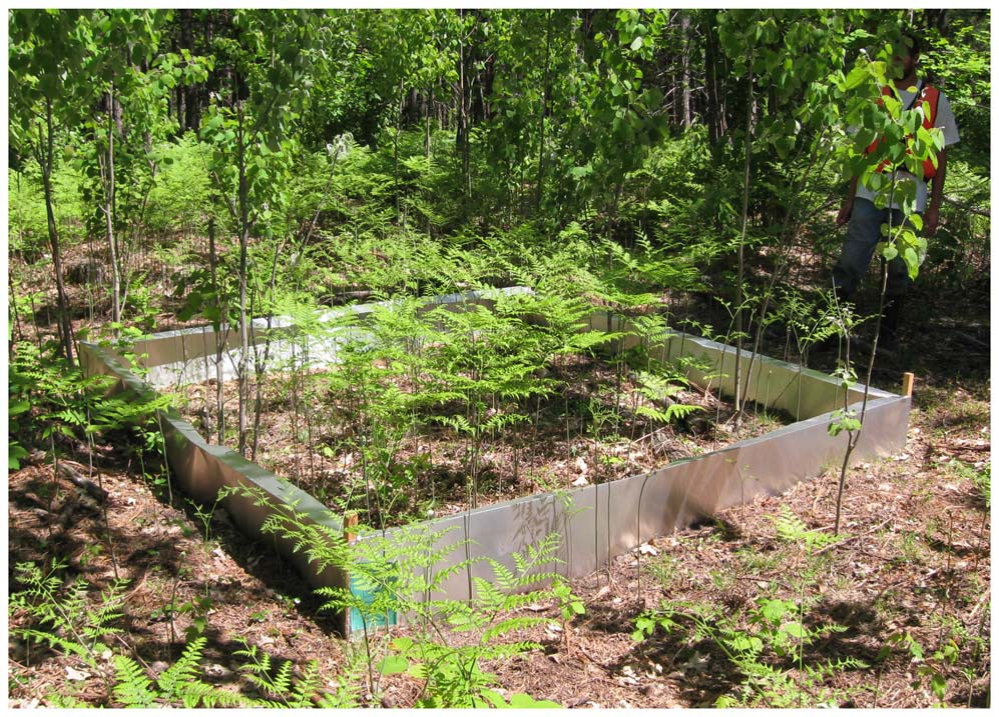
Example of a salamander field enclosure deployed in a harvested aspen stand.

In early May of 2010 and 2011 we erected a 9 m^2^ enclosure at the center of each 30×30 m lattice cell (total  =  45 cells). Enclosures were constructed of aluminum flashing 50 cm high and buried 12–15 cm into the ground (Figure 1). The top of each fence was bent inward at 90° to prevent salamander escape. We did not attempt to remove salamanders that naturally occurred within the enclosure area (*i.e.*, unmarked salamanders). CWD that extended beyond the enclosure boundary were cut and those portions external to the enclosure were removed prior to fence construction. We tested the effectiveness of the field enclosure design for preventing salamander escape over the top by adding 12 salamanders to a 0.09 m^2^ replica enclosure, placed in a covered plastic container with air holes, over a 3-day period. No salamanders escaped from the replica during this time. In 2010 we also visited field enclosures during warm, rainy nights to observe if salamanders were attempting to scale the wall; we never observed salamanders attempting to get in or out of the enclosures.

In mid-May, we added 10 adult salamanders to each enclosure that were individually marked with visual implant elastomer (VIE; Northwest Marine Technology, Shaw Island, Washington; [38]). These salamanders were captured within 1 km of our study stands and added to the enclosure within 24 h of capture. Release locations within an enclosure were randomly assigned. Marked salamander density within the enclosures (≈1.1 m^2^) was comparable to observed densities in mature forests in northern Michigan [39]. From mid-May to mid-June the average maximum temperature was 23.3°C (range 11.7 to 32.8°C).

In mid-June, we searched enclosures for salamanders on three successive visits, separated by 3.0±1.2 days (mean ± 1 standard deviation). We assumed that unmarked salamanders made habitat selection choices resulting in their occurrence within the enclosure prior to construction. Prior to each search, enclosures were gridded into 1 m^2^ sections to ensure searches were performed systematically. Observers thoroughly searched their assigned 1 m^2^ area by examining leaf litter and underneath and within pieces of CWD for salamanders. When performing searches, observers placed all leaf-litter and CWD in plastic bins that were assigned to each 1 m^2^ area until nothing remained inside the enclosure except for rooted herbaceous and woody vegetation, and mineral soil. Observers placed leaf-litter and CWD back into the enclosure after searching was complete and attempted to reconstruct the micro-habitat to pre-sampling conditions. Captured salamanders were held in coolers until the sampling event was complete. Two observers checked each salamander for unique VIE markings and then re-released them into the enclosure at the point of capture. Unmarked salamanders that were captured inside the enclosures were not marked.

We used N-mixture models [31] to estimate abundance (N*_i_*) and detection probabilities (p) of unmarked salamanders within the enclosures. Additional details regarding hypothesis and model development can be found in Text S1. Briefly, we fit models where abundance varied as a function of CANOPY above, and CWD within, each enclosure. Similar to the large-scale analysis, we treated CANOPY as a continuous variable (i.e., percent canopy cover) for all analyses. We also fit models where salamander abundance was allowed to vary between enclosures that were situated in 1–5 or 40–60 year-old forest stands (i.e., CONTROL covariate). Salamander detection probability was either held constant (p(.)), allowed to vary as a function of CWD within the enclosures (p(CWD)), or vary between our three sampling events (p(*t*)).

We used the Huggins parameterization of the robust design population model [40], [41] to estimate individual salamander survival (S*_i_*), initial capture (p*_t_*) and recapture probabilities (c*_t_*) of individually marked salamanders. Here, we used subscript “*i*” to denote that survival estimates apply to individual salamanders, as opposed to estimates from abundance models that apply to individual sites (“*I*”). Our robust design framework consisted of two primary periods: a salamander additions period and a capture/recapture period. During the additions period, marked salamanders were added to the enclosure, as described above. After one month, we searched through all enclosures for marked salamanders on three successive visits (*i.e.,* 3 secondary periods during primary period 2). Thus,
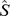
represents the probability that a marked salamander survived from mid-May (primary period 1) until mid-June (primary period 2) and was available for capture during primary period 2. Initial capture (p*_t_*) is the probability that a marked salamander was captured for the first time during visit *t* of the second primary period (*t* = 1, 2, 3). Recapture (c*_t_*) is the probability a marked salamander was recaptured during visit *t*, conditional on it being captured at least once before during a previous visit (note: c_1_ = 0).

Survival probability was allowed to vary as a function of CANOPY, CWD, or CONTROL (see Text S1 for additional model details). We explored whether capture and recapture probabilities were equal and constant across time (p(.)  =  c(.)), varied across our sampling events (p(*t*)  =  c(*t*)), varied as a function of CWD within an enclosure (p(CWD)  =  c(CWD), or if recapture probabilities were lower than initial capture probabilities (p(.)  =  c(.) + b).

### Data Analysis

We analyzed our abundance data using R (version 2.12.1, http://www.r-project.org/; R Development Core Team 2011) with the add-in package unmarked [42]. We analyzed survival data using Program MARK (MARK, version 5.1, http://www.cnr.colostate.edu/~gwhite/mark/mark.htm). We used Akaike's Information Criterion, adjusted for small sample size (AIC*_c_*), to rank models [43]. We used cumulative AIC weights (*w_+_*) and evaluation of 95% confidence intervals to determine relative importance of covariates and model parameters. We report model averaged estimates and unconditional 95% confidence intervals for all back-transformed parameters. We also conducted a Pearson Correlation Analysis to test for potential density dependent effects between counts of marked and unmarked salamanders within enclosures.

### Ethics Statement

Our salamander sampling and handling protocols were approved by the Michigan State University Animal Care and Use Committee (Animal Use Form no. 07/08-118-00).

## Results

For the 1 to 5 year-old stands, covariates CANOPY and CWD were weakly, negatively correlated in the large- (df  = 105, r = −0.11, *R^2^* = 0.01) and small-scale (df = 34, r = −0.33, *R^2^*  = 0.11) analyses.

### Large-scale Abundance

Salamander capture probability (p) ranged between 0.27–0.49 among all candidate models. Capture probability was positively related to the quantity of CWD along each transect (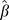 *_CWD_*  =  0.27 (95% CI: 0.07–0.47) for the top-ranking model; Table 1). Cumulative weight (*w_+_*) for models that included the effect of CWD on capture probability was 0.69 (Table S1). Our exploratory analysis revealed no support for the influence of average daily temperature or daily precipitation on salamander detection probability (Table S1). All weather covariates had confidence intervals that overlapped zero and the estimated effects of CANOPY and CWD on salamander abundance were not influenced by the inclusion of weather covariates on detection probability (Table S1). From here forward we report model results which lack exploratory weather covariates.

**Figure 2:**
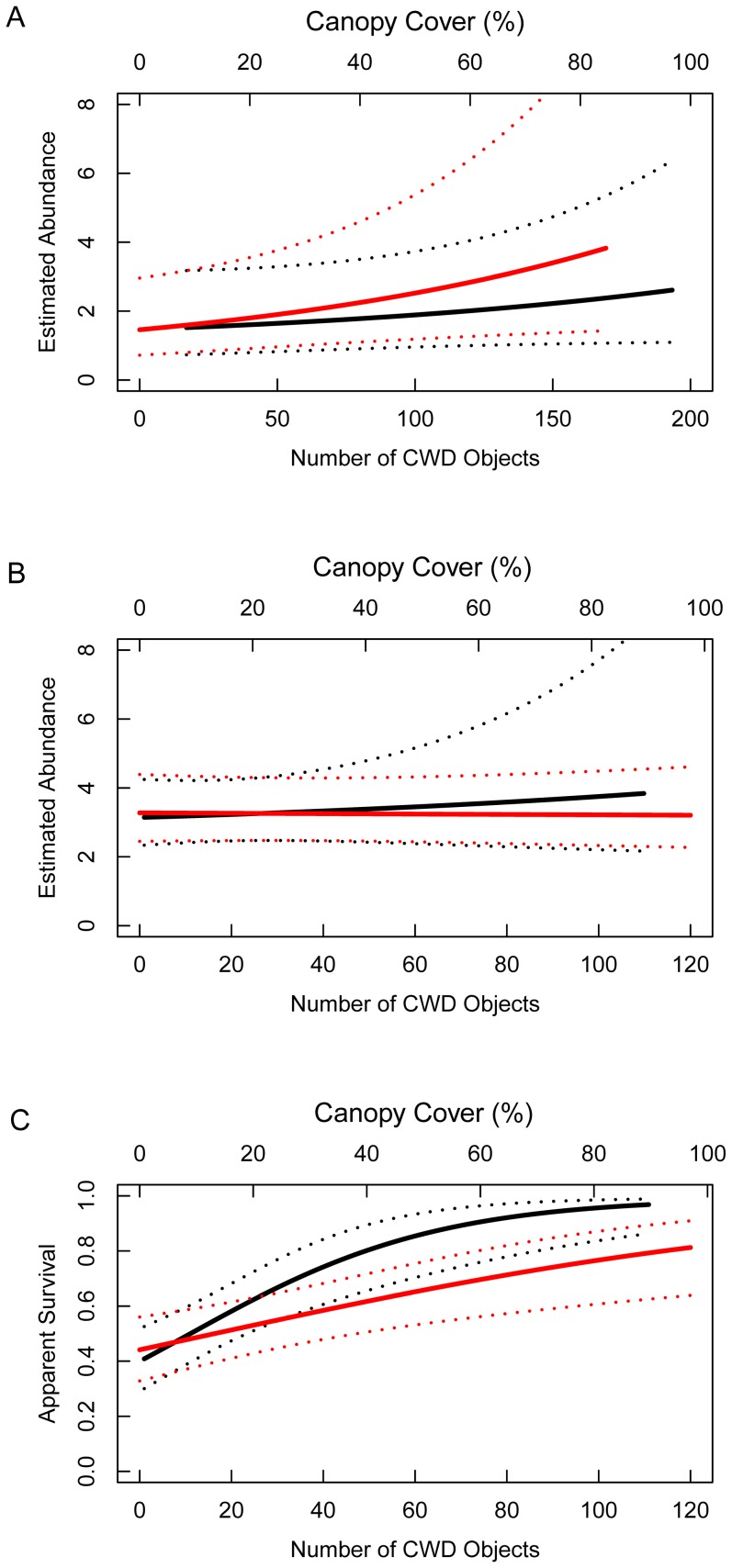
Salamander abundance and survival estimates in harvested forests. Abundance and apparent survival estimates for red-backed salamanders in 1–5 year-old clearcut aspen stands in the northern Lower Peninsula of Michigan, 2010–2011, explained as a function of the amount of green tree canopy retention (Canopy Cover  =  red line), and the number of coarse woody debris (CWD  =  black line) objects at each site. Dotted lines are 95% confidence intervals. A) Abundance estimates of unmarked salamanders at 3,600 m^2^ sites, B) abundance estimates of unmarked salamanders at 9 m^2^ sites, and C) apparent survival estimates of marked salamanders at 9 m^2^ sites. All estimates were generated using model averaging.

The mean standard deviation for salamander counts among 3 transects within a site was 0.36 (range 0.00–2.08), suggesting that variation in salamander counts among transects within a site was relatively low. As predicted, abundance estimates for red-backed salamanders were higher for 40–60 year-old, 3,600 m^2^ sites (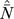 *_40-60yr Sites_*  =  4.8, 95% CI: 2.2–10.1) when compared to 1–5 year-old sites (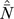 *_1-5yr Sites_*  =  1.7, 0.9–3.4). Mean values of percent canopy cover (CANOPY) and counts of CWD objects within clearcut sites were 15±17 (±1SD) and 69±32, respectively. Cumulative weights for all models that included the effects of CANOPY or CWD on salamander abundance were 0.77 and 0.50, respectively (Table S1). Large-scale salamander abundance was positively correlated with CANOPY and CWD (Table 1); however, the estimated effect sizes were imprecise for both covariates (Figure 2a). CANOPY was included in 3 of 4 of our top-ranking models. The 95% confidence intervals for CANOPY did not overlap zero for any of the top-ranking models (i.e., AIC*c* weight ≥10%) that included CANOPY (Table 1). CWD occurred in two of four top-ranking models and the 95% CI overlapped zero for one of those (Table 1). Collectively, the evidence suggests that salamander abundance was weakly related to the amount of CANOPY and CWD at 3,600 m^2^ sites within 1–5 year-old aspen stands.

### Small-scale Abundance

Estimated capture probabilities of unmarked salamanders in 9 m^2^ enclosures were 0.70 (0.53–0.83), 0.35 (0.26–0.46), and 0.28 (0.20–0.38) for our three sampling events, respectively. Evidence suggests that capture probability was not influenced by CWD count (Table 1). The 95% confidence intervals overlapped zero (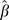 *_CWD_*  =  −0.21 (−0.51–0.09)) for the top model which included an association between CWD and capture probability.

On average, we detected 1.6 unmarked salamanders per 9 m^2^ enclosure, per visit (±2.2; 1 SD). Mean estimated abundance of unmarked salamanders was 5.6 (3.8–8.1; 95% CI) and 3.2 (2.6–4.2) for 40–60 and 1–5 year-old sites, respectively. Mean values of percent canopy cover (CANOPY) and counts of CWD objects within clearcut sites were 28±17 (±1SD) and 29±28, respectively. Cumulative weights for models that included the effects of structural retention covariates were 0.20 and 0.24 for CANOPY and CWD, respectively (Table S1). For all models, the 95% CIs overlapped zero for CANOPY and CWD (Table 1). Small-scale abundance of unmarked salamanders was not positively correlated with CANOPY or CWD within the enclosures (Figure 2b).

### Salamander Apparent Survival

Observers averaged 83 min (range  =  47–184) to search an enclosure during a sampling visit. Observers had 270 captures of marked salamanders during the three visits. Of these captures, 265 were made while searching underneath leaf-litter and CWD, and five were from salamanders found in the bottom of the plastic bins at the end of the sampling event. Within each enclosure, there was no relationship between the total number of unmarked salamanders captured and the total number of marked salamanders recaptured in mid-June (*R^2^* = 0.02).

**Figure 3:**
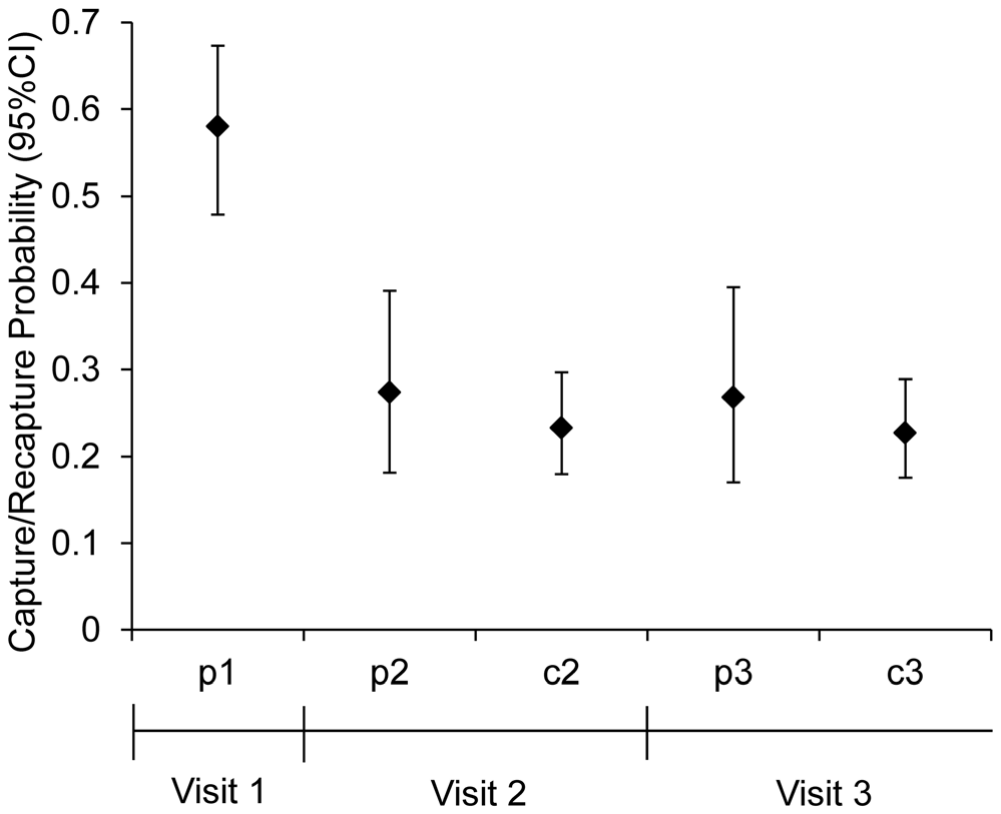
Salamander capture and recapture probabilities within field enclosures. Model-averaged estimates of initial capture (p) or recapture (c) probability of salamanders during three sampling events (visits) in mid-June, northern Lower Peninsula of Michigan, 2010–2011. Initial capture (p*_t_*) is the probability that a marked salamander is captured for the first time during visit *t*, conditional on it surviving and being available for capture. Recapture (c*_t_*) is the probability a marked salamander is recaptured during visit *t*, conditional on it being captured at least once before during a previous visit.

Initial capture probabilities of marked salamanders were approximately 50% lower in the second and third site visits compared to the first visit (Figure 3). The estimated probability of capturing a marked salamander at least once during the 3 visits was 0.78 (*i.e.,* 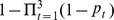). Model-averaged recapture probabilities were slightly lower than initial capture probabilities (Figure 3), but the effect was not strong as models with the capture probability structure p(*t*) = c(*t*) + b garnered only 0.29 of the cumulative model weight (Table S1). Capture and recapture probabilities were not influence by the amount of CWD within the enclosures (Table 1; 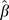 *_CWD_*  =  0.02 (−0.15–0.19) for top model which included CWD).

Excluding the effects of CANOPY and CWD, apparent survival estimates were 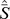  =  0.62 (0.50–0.72) and 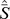  =  0.64 (0.46–0.79) for 1–5 and 40–60 year-old sites, respectively. Substantial evidence (*w_+_*>0.95 for CANOPY and CWD; Table S1) indicated that CANOPY and CWD positively influenced salamander apparent survival from mid-May until mid-June in 1–5 year-old harvest stands (Figure 2c). For our top-ranked model, S(CANOPY + CWD + CONTROL), p(*t*)  =  c(*t*) beta parameter estimates were 0.71 (95% CI: 0.26–1.17) and 0.96 (0.50–1.42) for CANOPY and CWD, respectively (Table 1). Estimated apparent survival probabilities were >0.80 for 1–5 year-old sites that contained high levels of canopy cover or CWD (Figure 2c).

## Discussion

Ultimately, population processes such as survival, reproduction, and movement determine patterns in species occupancy, richness, and abundance [21]. As such, occupancy and abundance are indirect representations of demographic processes and may offer limited insight into faunal response to habitat degradation [20], [21], [44]. An appealing characteristic of occupancy and abundance studies is that they can be conducted over broader spatial and shorter temporal scales, thereby increasing the spatial breadth of inference and potential applicability to management. Our inferences regarding salamander response to structural retention in aspen clearcuts were largely conditional on our population parameter of interest. Whereas we found limited support for an influence of structural retention on salamander abundance at two nested spatial scales, we found strong evidence for a positive effect of structural retention on apparent survival probability over 1 month in the summer.

### Does Structural Retention Promote Abundance?

At 3,600 m^2^ and 9 m^2^ we observed higher salamander abundances at 40–60 year-old sites compared to 1–5 year-old sites. This finding is consistent with other studies that show patterns in amphibian counts are positively correlated with time since timber harvest [9], [10], [34]. In our study, estimated salamander abundance was positively but weakly related to CANOPY and CWD quantity at the large scale (*i.e.,* 3,600 m^2^), but not at the small scale (*i.e.,* 9 m^2^). At the large spatial scale abundance estimates for 1–5 year-old sites were consistently >20% lower than 40–60 year-old sites, even among sites with high levels of structural retention. Other studies have shown that structural enhancement of young clearcuts can benefit amphibian occurrence and abundance under certain conditions [24]. Our multi-scaled abundance results suggest that the benefits are more uncertain than previously realized.

By sampling across a broad spatial extent (560,000 ha) and over 90 different timber harvest stands, our study represents a realistic range of forest stand conditions typically found in young aspen of the northwestern Lower Peninsula of Michigan. This is important because the spatial scales of past amphibian-forestry studies that assessed occupancy or abundance have often been limited to few experimental forest stands [19], [34], [45], [46]. Inferring broad-scale impacts based on limited spatial extent and site replication is a common limitation in research on the effects of timber harvest on forest biodiversity [24], [37], even though the potential pitfalls of doing so in amphibian-habitat research have been discussed [18].

### Does Structural Retention Promote Survival?

In contrast to our abundance results, our demographic study showed that leaving structural elements, such as vertical green trees and horizontal CWD within young harvest stands may ameliorate the negative impacts of clearcutting on amphibian survival over 1 month. Past research has shown that clearcutting negatively impacts amphibian survival [19], [47] (but see [48]). However, the role of structural retention for influencing demographic parameters is less clear [49]. An enclosure study by Rittenhouse et al. [50] showed that amphibian survival over 30 hours in clearcuts was higher when enclosures were deployed in brushpiles compared to enclosures deployed in open areas with no microhabitat refugia. Our study differs from Rittenhouse et al. [50] in both the area encompassed by the enclosure (0.07 m^2^; Rittenhouse et al. [50], 9 m^2^; our study) and the time over which survival was estimated (30 h; Rittenhouse et al. [50], ≈1 month; our study). Our survival study is temporally limited to 1 month during a relatively mild portion of the summer. It is unclear if salamander survival probability would continue to show a strong positive relationship with CANOPY and CWD during the hottest portions of the summer, July and August. Nonetheless, our research shows that various combinations of green-tree and CWD retention can be used to achieve high apparent survival probabilities for salamanders inhabiting aspen clearcuts in early summer.

Our survival analysis is based on the assumption of no temporary emigration. Although our enclosures prevented horizontal emigration, we were unable to account for potential vertical emigration of salamanders into the soil profile. Live salamanders that migrated into the soil profile before our first resampling event and remained there throughout our three replicate visits were effectively unavailable for capture. These salamanders would likely be recorded as false absences, which can lead to negative bias in survival estimates and potentially bias covariate effects if emigration rates were influenced by covariates that also influence survival [51]. Capture and recapture estimates from our survival study showed that salamanders within the enclosure were less likely to be detected during surveys two and three. This suggests that salamanders in our study had the ability to temporarily emigrate from the sampling area into the soil profile. Thus, our survival estimates likely possess some negative bias and should be considered apparent survival, where *S* is the product of true survival probability and the probability of remaining in the above ground, sampling area. However salamander capture and recapture probabilities did not depend on the quantity of CWD within the enclosures (Table S1). This suggests that the availability of CWD within the enclosure did not influence the probability that salamanders retreated underground and became unavailable for capture. Although our survival estimates may be biased low, the estimated effects of CWD on apparent survival are likely unbiased.

### Population Patterns vs. Process

Single pattern- or process-based response variables are often used to assess the impacts of anthropogenic disturbance on biological diversity; seldom are they jointly evaluated in the same ecological system [19], [52]. Inferential power of studies that directly assess demographic processes, such as survival, is generally greater than studies that assess demographic indices, such as occupancy or abundance; however, strength typically comes at the expense of inferential breadth [48]. Conducting detailed studies of demographic processes and population patterns is important for understanding broad-scale anthropogenic impacts and the results of management actions. Our study addresses these concerns through a joint evaluation of multi-scaled abundance patterns and small-scale demographic processes within an actively-managed forested landscape.

Our research shows that structural retention harvest, when done at relatively small spatial scales, can reduce the negative impacts of clearcutting on terrestrial salamanders by lowering apparent mortality rates in 1–5 year-old clearcuts during the summer. This provides resource managers with direct evidence that alternative forest management practices can positively influence salamander population dynamics. However, when viewed at larger spatial scales, we found minimal evidence that structural retention had a strong, positive influence on abundance at sites that were 1–5 years post-harvest. One potential explanation for these seemingly conflicting results is the difference in the temporal scales of abundance and survival studies. Our abundance studies represent snapshots of salamander populations that were subjected to anthropogenic disturbance events that occurred 1–5 years previous, whereas our survival study was conducted over one month. Thus, although structural retention may increase salamander survival over one month, it is unclear if this benefit is negated over a longer temporal period.

An alternative explanation for our conflicting results is that patterns in salamander abundance within clearcuts may be largely influenced by movement dynamics, rather than mortality. Some amphibians will emigrate from recently harvested forest stands (*i.e.,* evacuation hypothesis; [23], [53]). Thus, lower abundance estimates for our 1–5 year-old stands could reflect behavioral avoidance of salamanders to clearcuts, regardless of the local availability of structural retention. Indeed, salamander abundances at both spatial scales were not strongly correlated with CANOPY or CWD. This suggests the distribution of free-ranging salamanders across young forest stands was relatively uniform with respect to our habitat covariates. Similar to other past amphibian-forestry experiments, our survival study did not allow salamanders to choose particular treatments through habitat selection [48], [50]. In our survival study we released naïve salamanders into enclosures, thereby eliminating their opportunity to seek refuge in adjacent forest stands and forcing them to make habitat selection choices within a 9 m^2^ area. Although our enclosure experiment shows that salamanders can survive over a single season in clearcuts when habitat refugia are provided, it is unclear if these salamanders would choose to remain in clearcuts, if given the option [53], [54]. This explanation is consistent with the observed weak correlation between salamander abundance at two spatial scales and structural retention, and the strong effect of structural retention on salamander apparent survival.

## Conclusions

Regardless of potential mechanism(s), our study suggests that comprehensive conservation goals for terrestrial salamanders, and other forest-obligate species, will not be accomplished by simply retaining structure within individual harvest units. Our research shows that structural retention can be used to influence population vital rates of terrestrial salamanders, over a relatively short time span. However, our abundance analyses suggest that broad-scale conservation efforts should consider factors that likely extend beyond localized patches of retention. For example, if movement dynamics are truly important to the persistence of forest floor obligates, these species may also require provision of high quality habitats like late-successional forests with structurally complex understories in the managed landscape. Habitat degradation will continue as a dominant force in the global biodiversity and sustainability crisis for the foreseeable future [3], [14]. Hence, developing research and monitoring programs that assess broad-scale changes in population patterns, and the demographic processes underlying these changes, should be a shared goal of ecologists and resource managers alike.

## Supporting Information

Text S1 **Complete description of model development.**
(DOCX)

Table S1 **Complete model set for** **Table 1** **.** Ranking of candidate N-mixture (abundance) and Robust Design (survival) models for red-backed salamanders in harvested aspen stands in the northern Lower Peninsula of Michigan, USA, 2010–2011.
(DOCX)
