# Supplementary material for: Comparing Population Patterns to Processes: Abundance and Survival of a Forest Salamander following Habitat Degradation
Source: PLoS One. 2014 Apr 9;9(4):e93859. doi: 10.1371/journal.pone.0093859 (PMC3981728; doi:10.1371/journal.pone.0093859)
Supplement: Text S1 — Complete description of model development. (DOCX) [file pone.0093859.s001.docx]

*Large-scale Abundance Models*

To represent our hypotheses, we constructed models where salamander abundance (N) varied linearly on the log scale as a function of CANOPY within the 0.36 ha cell (log(N*_I_*) = β­_0_ + β_1_(CANOPY*_I_*)), or the cumulative number of CWD objects encountered during surveys along the three transects (log(N*_I_*) = β­_0_ + β_1_(CWD*_I_*)). We hypothesized that CANOPY and CWD could operate independently, or as additive effects on abundance (N*_I_* (CANOPY*_I_* + CWD*_I_*)). Although we stratified canopy cover into different categories during site selection, we treated CANOPY as a continuous variable for all analyses. We predicted that salamander detection probability would generally be constant across time, as all transects within a site were sampled at the same time and all sites were sampled within a single month (Otto et al. 2013, Ecography: 1299-1309). However, we were concerned that variable quantities of downed wood along transects could influence the ability of our observers to detect salamanders. Hence, we constructed a model where detection probability (*p*) varied as a function of CWD encountered along transect *j* (CWD*_j_*; logit(*p_j_*) = β­_2_ + β_3_(CWD*_j_*). We note the distinction between CWD*_I_*, a site covariate, which is the total number of cover objects encountered among all 3 surveyed transects within a site, and CWD*_j_*, a detection covariate, which is the number of cover objects along transect *j* within a site (*j* = 1,2,3). Additive combinations of abundance and detection covariates resulted in 8 candidate models.

We also conducted an exploratory analysis on the influence of daily precipitation (PRECIP*_d_* and daily maximum temperature (TEMP*_d_*) on salamander detection probability across our sampling dates. We included TEMP and PRECIP as additional detection covariates in our 2 top-ranking models from our primary analysis. We calculated averages for daily maximum temperature and daily precipitation among 5 weather stations located within our 4 county sampling area. Weather data were obtained from the National Oceanic and Atmospheric Administration (<http://www.nws.noaa.gov/climate/xmacis.php?wfo=apx>; accessed August 2011). This resulted in 4 exploratory models. Results are reported in Appendix 2.

*Small-scale Abundance Models*

Similar to our large-scale abundance analysis, we constructed models that allowed abundance of unmarked salamanders (N*_I_*) to vary as a function of CANOPY, CWD and CONTROL. We hypothesizes that these covariates could act independently or as additive effects on abundance (N*_I_*(CANOPY*_I_ +* CWD*_I_* + CONTROL*_I_*)). Salamander detection probability was either held constant (p(.)), allowed to vary as a function of CWD within the enclosure (p(CWD), or vary between our three sampling events (p(*t*)). We considered independent and additive effects of p(*t*) and p(CWD). We included the p(*t*) model because previous work suggests salamanders may temporarily emigrate from sites following habitat disturbances caused by sampling (Otto et al. 2013, Ecography: 1299-1309). Although our enclosures prevented horizontal emigration, they could not prevent vertical emigration into the soil profile. Thus, we would expect lower detection probabilities in the second and third sampling events if salamanders emigrated underground following the first sampling event.

By including p(CWD) model structure, we were able to determine if detection probability was lower at sites with reduced availability of CWD. Strong support for p(CWD) would suggest that temporary emigration was influenced by the amount of woody refugia within the enclosures. Additive combinations of abundance and detection covariates resulted in 16 candidate models.

*Robust Design (Survival) Models*

We hypothesized that salamander survival probability in 40-60 year-old sites would be higher than survival in 1-5 year-old sites, given the literature suggesting that amphibians are negatively impacted shortly after timber harvest (*e.g.,* Semlitsch et al., 2009, Bioscience: 853-862). We also predicted that salamanders within enclosures with higher quantities of CWD would exhibit higher survival rates because these structures trap moisture on the forest floor and may reduce risk of amphibian desiccation (Rittenhouse et al., 2008, Copeia: 807-814). We modeled CWD quantity as a continuous covariate on salamander survival (logit (S*_i_*) = β­_0_+ β_1_ × CWD*_e_*) where CWD*_e_* equals the total number of CWD objects within the enclosure which contained salamander *i*. We also hypothesized that salamander survival would be higher within enclosures that were shaded by retained green-trees. To represent this hypotheses, we modeled CANOPY as a continuous covariate on salamander survival (logit(S*_i_*) = β­_2_+ β_3_ × CANOPY*_e_*) where CANOPY equaled the amount of canopy cover within the 30 × 30 m cell that contained salamander *i*. As with the abundance analysis, we modeled survival probability as a function of a single covariate only (S(CANOPY) or S(CWD)), or as an additive effect (S(CANOPY + CWD)).

We explored whether capture and recapture probabilities were equal and constant across time (p(.)) or if these probabilities decreased across our sampling events (p(*t*) = c(*t*)). Support for the latter model structure is based on previous work which suggests salamanders temporarily emigrate from sites following habitat disturbances caused by sampling (Otto et al. 2013, Ecography: 1299-1309). We note the distinction between emigration that occurs between primary periods (“γ” in traditional Robust Design models), which we fixed at “0”, and temporary emigration that occurs within a primary period, between surveys. Although our enclosures restricted horizontal movements of salamanders, temporary unavailability could still occur if salamanders moved vertically into the subterranean environment following a sampling disturbance. We fit models to test whether the probability of initial capture and recapture were affected by the quantity of CWD within each enclosure (p(CWD) = c(CWD). Our final hypothesis was based on the prediction that salamanders may exhibit a behavioral response to being captured, thereby resulting in lower recapture probability during subsequent surveys (*i.e.,* trap response). We represented this hypothesis by including an additive constant on recapture probability, which allowed initial capture and recapture probabilities to be different (p(.) = c(.) + b). Additive combinations of abundance and detection covariates resulted in 32 candidate models.
