# Supplementary material for: Comparing Population Patterns to Processes: Abundance and Survival of a Forest Salamander following Habitat Degradation
Source: PLoS One. 2014 Apr 9;9(4):e93859. doi: 10.1371/journal.pone.0093859 (PMC3981728; doi:10.1371/journal.pone.0093859)
Supplement: Table S1 — Complete model set for Table 1 . Ranking of candidate N-mixture (abundance) and Robust Design (survival) models for red-backed salamanders in harvested aspen stands in the northern Lower Peninsula of Michigan, USA, 2010–2011. (DOCX) [file pone.0093859.s002.docx]

| Model | Δ AIC_c_^a^ | *w*^a^ | *K*^a^ | −2*l*^a^ | CANOPY ^b^ | CWD ^b^ |
| --- | --- | --- | --- | --- | --- | --- |
|  |  |  |  |  |  |  |
| Large-Scale Abundance |  |  |  |  |  |  |
| N(CANOPY + CONTROL), p(CWD) | 0.00 | 0.36 | 5 | 558.8 | 0.21 (0.03-0.40) |  |
| N(CANOPY + CWD + CONTROL), p(.) | 1.18 | 0.20 | 5 | 559.9 | 0.21 (0.02-0.40) | 0.23 (0.06-0.40) |
| N(CANOPY + CWD + CONTROL), p(CWD) | 1.40 | 0.18 | 6 | 558.0 | 0.21 (0.03-0.40) | 0.11 (-0.13-0.35) |
| N(CONTROL), p(CWD) | 2.55 | 0.10 | 4 | 563.5 |  |  |
| N(CWD + CONTROL), p(.) | 3.39 | 0.07 | 4 | 564.3 |  |  |
| N(CWD + CONTROL), p(CWD) | 3.76 | 0.05 | 5 | 562.5 |  |  |
| N(CANOPY + CONTROL), p(.) | 5.00 | 0.03 | 4 | 565.9 |  |  |
| N(CONTROL), p(.) | 6.99 | 0.01 | 3 | 570.0 |  |  |
|  |  |  |  |  |  |  |
| Exploratory Analysis: Large-Scale Abundance with Weather Covariates |  |  |  |  |  |  |
| N(CANOPY + CONTROL), p(CWD) | 0.00 | 0.21 | 5 | 558.8 | 0.21 (0.03-0.40) |  |
| N(CANOPY + CONTROL), p(CWD + PRECIP) | 0.43 | 0.17 | 6 | 557.0 | 0.23 (0.05-0.42) |  |
| N(CANOPY + CWD + CONTROL), p(.) | 1.18 | 0.12 | 5 | 559.9 | 0.21 (0.02-0.40) | 0.23 (0.06-0.40) |
| N(CANOPY + CWD + CONTROL), p(PRECIP) | 1.39 | 0.11 | 6 | 557.9 | 0.23 (0.04-0.42) | 0.26 (0.08-0.44) |
| N(CANOPY + CWD + CONTROL), p(CWD) | 1.40 | 0.11 | 6 | 558.0 | 0.21 (0.03-0.40) | 0.11 (-0.13-0.35) |
| N(CANOPY + CONTROL), p(CWD + TEMP) | 1.99 | 0.08 | 6 | 558.5 | 0.22 (0.04-0.40) |  |
| N(CONTROL), p(CWD) | 2.55 | 0.06 | 4 | 563.5 |  |  |
| N(CANOPY + CWD + CONTROL), p(TEMP) | 3.14 | 0.04 | 6 | 559.7 | 0.22(0.03-0.41) | 0.24(0.06-0.41) |
| N(CWD + CONTROL), p(.) | 3.39 | 0.04 | 4 | 564.3 |  | 0.24(0.05-0.42) |
| N(CWD + CONTROL), p(CWD) | 3.76 | 0.03 | 5 | 562.5 |  |  |
| N(CANOPY + CONTROL), p(.) | 5.00 | 0.02 | 4 | 565.9 |  |  |
| N(CONTROL), p(.) | 6.99 | 0.01 | 3 | 570.0 |  |  |
|  |  |  |  |  |  |  |
| Small-Scale Abundance |  |  |  |  |  |  |
| N(CONTROL), p(*t*) | 0.00 | 0.36 | 5 | 440.8 |  |  |
| N(CONTROL), p(*t* + CWD) | 0.94 | 0.23 | 6 | 439.0 |  |  |
| N(CWD + CONTROL), p(*t* + CWD) | 2.50 | 0.10 | 7 | 437.8 |  | 0.02 (-0.19-0.58) |
| N(CWD + CONTROL), p(*t*) | 2.65 | 0.10 | 6 | 440.7 |  | -0.01 (-0.21-0.18) |
| N(CANOPY + CONTROL), p(*t*) | 2.66 | 0.10 | 6 | 440.7 | -0.01(-0.20-0.18) |  |
| N(CANOPY + CONTROL), p(t + CWD) | 3.59 | 0.06 | 7 | 438.9 | -0.04(-0.23-0.15) |  |
| N(CANOPY + CWD + CONTROL), p(t) | 5.44 | 0.02 | 7 | 440.7 |  |  |
| N(CANOPY + CWD + CONTROL), p(t + CWD) | 5.45 | 0.02 | 8 | 437.8 |  |  |
| N(CONTROL), p(.) | 55.29 | 0.00 | 3 | 501.0 |  |  |
| N(CONTROL), p(CWD) | 56.88 | 0.00 | 4 | 500.2 |  |  |
| N(CWD + CONTROL), p(CWD) | 57.46 | 0.00 | 5 | 498.2 |  |  |
| N(CWD + CONTROL), p(.) | 57.65 | 0.00 | 4 | 501.0 |  |  |
| N(CANOPY + CONTROL), p(.) | 57.70 | 0.00 | 4 | 501.0 |  |  |
| N(CANOPY + CONTROL), p(CWD) | 59.36 | 0.00 | 5 | 500.1 |  |  |
| N(CANOPY + CWD + CONTROL), p(CWD) | 60.13 | 0.00 | 6 | 498.2 |  |  |
| N(CANOPY + CWD + CONTROL), p(.) | 60.18 | 0.00 | 5 | 501.0 |  |  |
|  |  |  |  |  |  |  |
|  |  |  |  |  |  |  |
| Survival |  |  |  |  |  |  |
| S(CWD + CANOPY + CONTROL), p(t) = c(t) | 0.00 | 0.56 | 7 | 1220.4 | 0.71 (0.26-1.17) | 0.96 (0.50-1.42) |
| S(CWD + CANOPY + CONTROL), p(t) = c(t) + b | 1.65 | 0.24 | 8 | 1219.1 | 0.67 (0.27-1.07) | 0.85 (0.44-1.27) |
| S(CWD + CANOPY + CONTROL), p(t + CWD) = c(t + CWD) | 2.91 | 0.13 | 8 | 1220.3 | 0.72 (0.26-1.18) | 0.94 (0.45-1.43) |
| S(CWD + CANOPY + CONTROL), p(t + CWD) = c(t + CWD) + b | 4.79 | 0.05 | 9 | 1219.1 |  |  |
| S(CWD + CONTROL), p(t) = c(t) | 8.65 | 0.01 | 6 | 1231.9 |  |  |
| S(CWD + CANOPY + CONTROL), p(.) = c(.) + b | 9.67 | 0.00 | 6 | 1232.9 |  |  |
| S(CWD + CONTROL), p(t) = c(t) + b | 11.25 | 0.00 | 7 | 1231.7 |  |  |
| S(CWD + CONTROL), p(t + CWD) = c(t + CWD) | 11.42 | 0.00 | 7 | 1231.8 |  |  |
| S(CWD + CANOPY + CONTROL), p(CWD) = c(CWD) + b | 12.47 | 0.00 | 7 | 1232.9 |  |  |
| S(CWD + CONTROL), p(t + CWD) = c(t + CWD) + b | 14.22 | 0.00 | 8 | 1231.7 |  |  |
| S(CONTROL), p(t + CWD) = c(t + CWD) + b | 15.48 | 0.00 | 5 | 1241.4 |  |  |
| S(CWD + CONTROL), p(.) = c(.) + b | 21.49 | 0.00 | 5 | 1247.4 |  |  |
| S(CANOPY + CONTROL), p(t + CWD) = c(t + CWD) + b | 22.23 | 0.00 | 8 | 1239.7 |  |  |
| S(CANOPY + CONTROL), p(t + CWD) = c(t + CWD) | 22.48 | 0.00 | 7 | 1242.9 |  |  |
| S(CWD + CONTROL), p(CWD) = c(CWD) + b | 24.15 | 0.00 | 6 | 1247.4 |  |  |
| S(CONTROL), p(t + CWD) = c(t + CWD) | 24.31 | 0.00 | 6 | 1247.5 |  |  |
| S(CONTROL), p(t)= c(t) + b | 26.41 | 0.00 | 5 | 1252.3 |  |  |
| S(CANOPY + CONTROL), p(t) = c(t) | 27.31 | 0.00 | 6 | 1250.5 |  |  |
| S(CONTROL), p(t) = c(t) | 27.56 | 0.00 | 5 | 1253.5 |  |  |
| S(CANOPY + CONTROL), p(t) = c(t) + b | 29.94 | 0.00 | 7 | 1250.4 |  |  |
| S(CANOPY + CONTROL), p(.) = c(.) + b | 41.26 | 0.00 | 5 | 1267.2 |  |  |
| S(CONTROL), p(.) = c(.) + b | 42.18 | 0.00 | 4 | 1270.6 |  |  |
| S(CANOPY + CONTROL), p(CWD) = c(CWD) + b | 43.37 | 0.00 | 6 | 1266.6 |  |  |
| S(CONTROL), p(CWD) = c(CWD) + b | 44.26 | 0.00 | 5 | 1270.2 |  |  |
| S(CWD + CANOPY + CONTROL), p(.) = c(.) | 72.02 | 0.00 | 5 | 1297.9 |  |  |
| S(CWD + CANOPY + CONTROL), p(CWD) = c(CWD) | 75.61 | 0.00 | 6 | 1298.8 |  |  |
| S(CWD + CONTROL), p(.) = c(.) | 81.18 | 0.00 | 4 | 1309.6 |  |  |
| S(CWD + CONTROL), p(CWD) = c(CWD) | 83.27 | 0.00 | 5 | 1309.2 |  |  |
| S(CANOPY + CONTROL), p(CWD) = c(CWD) | 90.89 | 0.00 | 5 | 1316.8 |  |  |
| S(CONTROL), p(CWD) = c(CWD) | 92.99 | 0.00 | 4 | 1321.4 |  |  |
| S(CANOPY + CONTROL), p(.) = c(.) | 98.54 | 0.00 | 4 | 1327.0 |  |  |
| S(CONTROL), p(.) = c(.) | 98.77 | 0.00 | 3 | 1329.6 |  |  |

^a^  ΔAIC*_c_* = difference from the Akaike’s Information Criterion (AIC) best model, adjusted for small sample size, *w* = AIC*_c_* model weight, *K* = no. of parameters, −2*l* = twice the negative log-likelihood.

^b^ Beta estimates for abundance covariates CANOPY and CWD with 95% CI in parentheses
